# Isolation of mitochondria from *Saccharomyces cerevisiae* using magnetic bead affinity purification

**DOI:** 10.1371/journal.pone.0196632

**Published:** 2018-04-26

**Authors:** Pin-Chao Liao, Istvan R. Boldogh, Stephanie E. Siegmund, Zachary Freyberg, Liza A. Pon

**Affiliations:** 1 Department of Pathology and Cell Biology, Columbia University, New York, NY, United States of America; 2 Department of Neurology, Columbia University, New York, NY, United States of America; 3 Department of Psychiatry, University of Pittsburgh, Pittsburgh, PA, United States of America; 4 Department of Cell Biology, University of Pittsburgh, Pittsburgh, PA, United States of America; National Institute of Environmental Health Sciences, UNITED STATES

## Abstract

Isolated mitochondria are widely used to study the function of the organelle. Typically, mitochondria are prepared using differential centrifugation alone or in conjunction with density gradient ultracentrifugation. However, mitochondria isolated using differential centrifugation contain membrane or organelle contaminants, and further purification of crude mitochondria by density gradient ultracentrifugation requires large amounts of starting material, and is time-consuming. Mitochondria have also been isolated by irreversible binding to antibody-coated magnetic beads. We developed a method to prepare mitochondria from budding yeast that overcomes many of the limitations of other methods. Mitochondria are tagged by insertion of 6 histidines (6xHis) into the *TOM70* (Translocase of outer membrane 70) gene at its chromosomal locus, isolated using Ni-NTA (nickel (II) nitrilotriacetic acid) paramagnetic beads and released from the magnetic beads by washing with imidazole. Mitochondria prepared using this method contain fewer contaminants, and are similar in ultrastructure as well as protein import and cytochrome c oxidase complex activity compared to mitochondria isolated by differential centrifugation. Moreover, this isolation method is amenable to small samples, faster than purification by differential and density gradient centrifugation, and more cost-effective than purification using antibody-coated magnetic beads. Importantly, this method can be applied to any cell type where the genetic modification can be introduced by CRISPR or other methods.

## Introduction

Isolated yeast mitochondria are used to study fundamental processes including mitochondrial respiration, metabolic activity, protein import and membrane fusion as well as interactions of mitochondria with the cytoskeleton, nuclear encoded mRNAs and other organelles. Analysis of the mitochondrial proteome, phosphoproteome, and lipidome all rely on preparation of highly purified mitochondria. Finally, isolated mitochondria are also utilized to investigate the assembly and interactions of mitochondrial protein complexes [1–8]. For many of these studies, crude mitochondria are isolated by differential centrifugation [9]. Although mitochondria isolated by this method contain contaminants, this method is widely used. To reduce contamination, crude mitochondria can be further purified by density gradient ultracentrifugation [10, 11]. However, this method is time-consuming, which can result in a decline in mitochondrial function, and it requires large amounts of starting material as well as expensive equipment.

Magnetic beads coated with antibodies have been used to isolate or deplete cells from mixed cell populations [12]. This method has been modified for isolation of mitochondria from mammalian cells [13], mouse tissues [14], and human cortex [15]. Recently, lipophilic and delocalized triphenyl phosphonium (TPP) cation-bound magnetic beads that recognize mitochondria by mitochondrial membrane potential (Δψ) have been used to isolate the organelle [16, 17]. These magnetic bead-based methods provide an easy and quick way to prepare mitochondria from small samples without ultracentrifugation. However, antibody-coated magnetic beads are expensive and TPP-magnetic beads select for mitochondria with high Δψ, which may not be representative of all mitochondria in cells or tissues. In addition, isolated mitochondria are irreversibly bound to antibody-coated or TPP magnetic beads, which may affect mitochondrial ultrastructure and/or cover epitopes on the mitochondrial outer membrane.

Here, we took advantage of the power of yeast genetics and existing magnetic bead-based methods for mitochondrial preparation to develop a simple and low-cost method to prepare highly purified mitochondria from small samples of yeast without ultracentrifugation. Mitochondria isolated using this method contain fewer contaminants compared to crude mitochondria isolated using differential centrifugation. Importantly, these mitochondria are intact and similar in ultrastructure, protein import and cytochrome c oxidase complex activity compared to crude mitochondria.

## Materials and methods

### Yeast growth conditions and strain construction

All *S*. *cerevisiae* strains were derived from the wild-type strain BY4741 (*MATa his3Δ1 leu2 Δ0 met15Δ0 ura3Δ0*) (Open Biosystems, Huntsville, AL). To measure yeast growth rates, cells were grown in glucose-based rich media (yeast extract/peptone/dextrose, YPD). For experiments in which mitochondrial redox state was measured using mito-roGFP1, cells were grown to mid-logarithmic phase (optical density OD_600_ = 0.1–0.3) in synthetic complete medium without uracil (SC-Ura). For mitochondrial isolation, yeast were grown using lactate-based medium. In all experiments, cells were cultured at 30°C.

To insert 6xHis into the *TOM70* gene at its chromosomal locus, a PCR cassette containing sequences that are homologous to regions upstream and downstream of the *TOM70* stop codon and flank sequences encoding 6xHis and the kanamycin resistance marker *KanMX6*, was amplified from pFA6a-6xGly-His-tag-KanMX6 (Addgene, Cambridge, MA) [18] using the forward primer 5’-TCAAGAAACTTTAGCTAAATTACGCGAACAGGGTTTAATGCGGATCCCCGGGTT-AATTAA-3’ and the reverse primer 5’-TTGTCTTCTCCTAAAAGTTTTTAAGTTTATGTTTACTG-TGAATTCGAGCTCGTTTAAAC-3’. Wild type cells were transformed with this PCR product using the lithium acetate method [19] and transformants were selected by growth on G418-containing YPD plates. Insertion of the tag was confirmed by sequencing using the following primer: 5’-GCCATTACTTTTGCTGAAGCCG-3’.

### Growth rate analysis

Mid-log phase (OD_600_ = 0.5) yeast were diluted to OD_600_ = 0.07. 10 μl of a diluted culture was added to 200 μl of YPD medium in a 96-well flat-bottom plate (Corning, Corning, NY), and the optical density of the culture (OD_600_) was measured every 20 min for 3 days using a plate reader (Tecan Infinite M200, Research Triangle Park, NC). Each strain was plated in quintuplicate and the growth curves averaged or maximum growth rate (slope) calculated using the greatest change in OD_600_ over a 240-min interval in 72 hrs. Growth rates were estimated using linear regression using Magellan software.

### Mito-roGFP1 imaging

Mito-roGFP1 imaging was performed using a modification of a previously described method [20–22]. Briefly, cells were transformed with a plasmid bearing the mito-roGFP1 sequence using the lithium acetate method and the transformants were selected on SC-Ura plates. Cells containing mito-roGFP1 plasmids were grown in SC-Ura medium to mid-log phase. Imaging was performed on an Axioskop 2 microscope (Zeiss, Thornwood, NY) equipped with a 100x/1.4 NA Plan-Apochromat objective and an Orca 1 cooled charge-coupled device (CCD) camera (Hamamatsu Photonics, Hamamatsu City, Japan) using excitation by an LED light source (CoolLED pE-4000, Andover, UK) at 365 and 470 nm for the oxidized and reduced form, respectively. Images were collected through the entire cell depth with 21 z-sections at 0.3-μm intervals and were deconvolved using a constrained iterative restoration algorithm with the following parameters: 507nm excitation wavelength, 60 iterations, 100% confidence limit (Volocity, Perkin-Elmer, Waltham MA). After subtracting background and thresholding, the reduced/oxidized ratio was calculated in Volocity software.

### Preparation of mitochondria using magnetic beads

Preparation of mitochondrial fractions for affinity purification using magnetic beads was carried out using a modification of a previously described method [11]. Cells expressing 6xHis-tagged Tom70 were grown to mid-log phase in lactate media for 16 hr at 30°C with aeration. Cells were then concentrated by centrifugation at 1,500 x g for 5 min at 4°C. The cell pellet was resuspended in water (50 ml/g yeast wet weight) and subjected to another round of centrifugation (1,500 x g for 5 min at 4°C). The supernatant from this wash was discarded and the weight of the “wet” cell pellet was determined.

**Fig 1:**
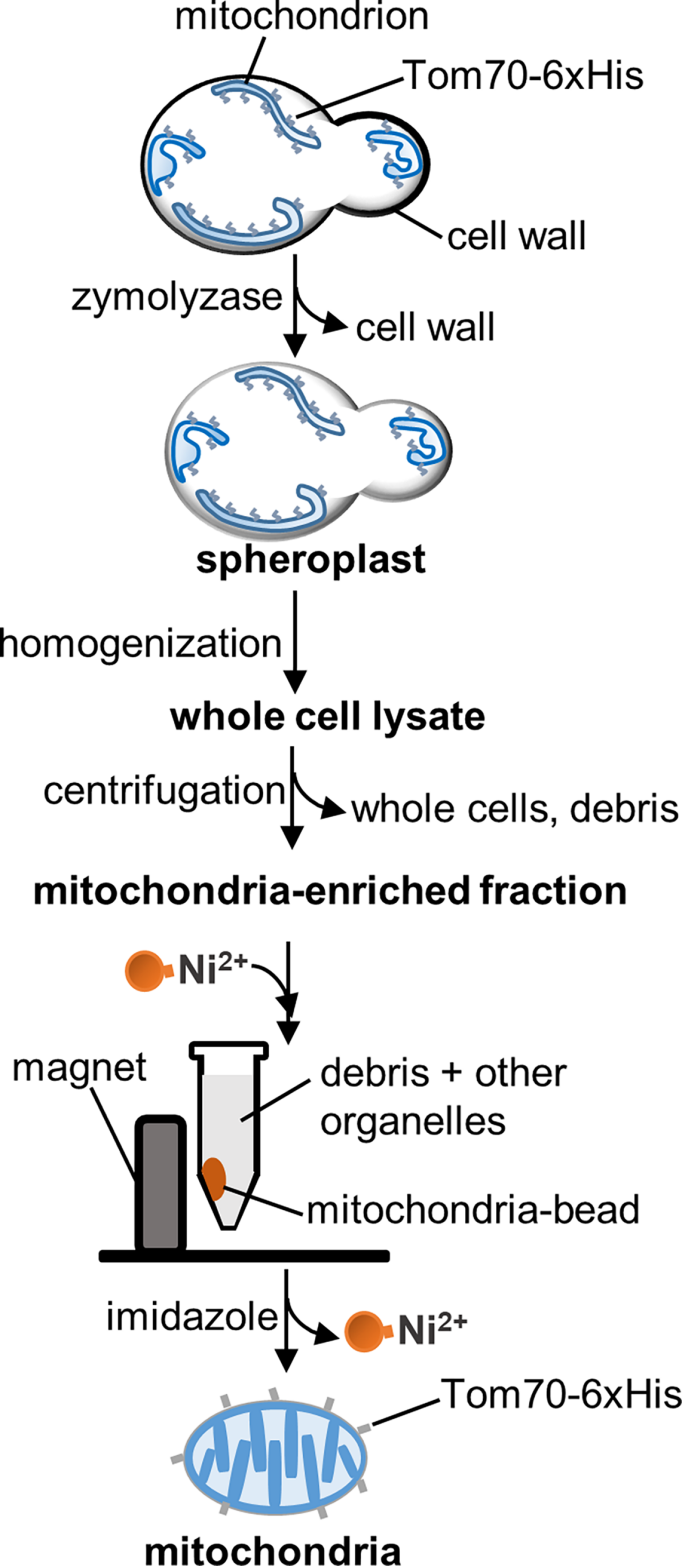
Scheme of mitochondrial isolation from yeast using magnetic beads. Yeast cells expressing Tom70-6xHis are converted to spheroplasts by incubation with Zymolyase, and disrupted using a Dounce homogenizer. The resulting whole-cell lysate is subjected to low-speed centrifugation to remove intact cells, cellular debris and nuclei, followed by high-speed centrifugation of the supernatant to concentrate mitochondria. We refer to the resuspended pellet from the high-speed centrifugation as the mitochondria-enriched fraction. The mitochondria-enriched fraction is the starting point for further purification of mitochondria. For affinity purification, the mitochondria-enriched fraction is incubated with HisPur Ni-NTA magnetic beads. Bead-bound mitochondria are separated from debris and other organelles in a magnetic field and eluted from beads with 500 mM imidazole.

To remove the cell wall, yeast cells were incubated in Tris-DTT buffer (0.1M Tris-SO_4_, pH 9.4 and 10 mM DTT) (5 ml/g yeast wet weight) for 15 min at 30°C with shaking, washed 1 time with SP buffer (1.2 M Sorbitol and 20 mM KPi, pH 7.4) (5 ml/g yeast wet weight) and incubated with Zymolyase 20T (Seikagaku Corporation, Tokyo, Japan) (7.5 mg/g yeast wet weight) in SP buffer (5 ml/g yeast wet weight) at 30°C for 40 min with shaking (Fig 1). Spheroplasts were concentrated by centrifugation at 4,500 x g at 4°C for 5 min, washed with ice-cold SEH buffer (0.6 M Sorbitol, 20 mM HEPES-KOH, pH 7.4, 2 mM MgCl_2_) (5 ml/g yeast wet weight), and resuspended in ice-cold SEH buffer containing a protease inhibitor cocktail (PI-1: 0.5-mg/ml Pepstatin A, 0.5 μg/ml Chymostatin, 0.5 μg/ml Antipain, 0.5 μg/ml Leupeptin, and 0.5 μg/ml Aprotinin; PI-2: 10 μM Benzamidine–HCl and 1 μg/ml 1,10-Phenanthroline; 1 mM PMSF) (5 ml/g yeast wet weight).

Spheroplasts were then homogenized using 15 forceful strokes of a pre-chilled glass/glass Dounce homogenizer (Wheaton Science Products, Millville, NJ). The homogenate was subjected to low-speed centrifugation (1,500 x g) for 5 min at 4°C, and the supernatant obtained was subjected to high-speed centrifugation (12,000 x g) for 10 min at 4°C. The resulting pellet was resuspended in ice-cold SEH buffer containing protease inhibitor cocktails to 1 ml/g yeast wet weight. We refer to this fraction as the “mitochondria-enriched fraction” (Fig 1).

Mitochondria were isolated from the mitochondria-enriched fraction using Ni-NTA magnetic beads (HisPur™ Ni-NTA Magnetic Beads, Thermo Scientific, Grand Island, NY). 100 μl of beads (1.25 mg of beads) was used for 1 ml of mitochondria-enriched fraction. The beads were first washed as follows: beads were transferred to a 1.5 ml microcentrifuge tube. The tube was placed in a Magnetic Separation Rack (6-Tube Magnetic Separation Rack, New England Biolabs, Ipswich, MA) and allowed to stand in the separation rack for 30 sec at room temperature (RT). This resulted in the concentration of magnetic beads at the lateral surface of the tube adjacent to the magnet in the separation rack. Residual liquid was removed using a micropipette. To wash the beads, the microcentrifuge tube was removed from the separation rack and 500 μl of SEH buffer was added. The tube was returned to the rack and SEH buffer was removed using a micropipette.

To bind mitochondria to the beads, 1 ml of the mitochondria-enriched fraction was added to the washed magnetic beads and incubated with the beads for 10–60 min at 4°C with gentle rotation. The mixture was then placed in the separation rack for 1 min at RT. The magnetic bead-bound mitochondria were then washed 3 times with 15 mM imidazole in ice-cold SEH buffer as described above. Mitochondria were eluted from the magnetic beads by incubation with 50 μl of 500 mM imidazole in ice-cold SEH buffer for 5 min with rotation at 4°C (Fig 1). The tube was again placed in the magnetic field to trap the beads. The released mitochondria were collected with a micropipette, concentrated by centrifugation at 12,000 x g for 5 min at 4°C and resuspended in ice cold SEH buffer, as needed.

The protocol described is for preparation of mitochondria from mid-log phase yeast propagated in 1.5 L of lactate medium. The wet weight of yeast grown under these conditions is typically 4 g. However, this method has also been used to prepare mitochondria from 250-ml cultures in lactate medium. Yields of bead-purified mitochondria vary depending on the amount of starting material and the time of incubation of the magnetic beads with the mitochondria-enriched fraction. We typically obtain 100–130 μg of mitochondria from 1 g of wet yeast after incubation of the mitochondria-enriched fraction with the magnetic beads for 60 min. The yield obtained after 10-min incubation is reduced by 25% compared to that obtained after a 60-min incubation, but is sufficient for most biochemical assays.

### Isolation of crude mitochondria

The mitochondria-enriched fraction was prepared as described above. To further remove debris, 1 ml of mitochondria-enriched fraction was subjected to 2 rounds of low-speed centrifugation (700 x g for 5 min and 1,500 x g for 5 min) at 4°C using a benchtop microcentrifuge. The supernatant obtained was subjected to high-speed centrifugation (12,000 x g for 10 min at 4°C), and the pellet obtained was resuspended in ice-cold SEH buffer, as needed. We refer to this fraction as “crude mitochondria”. The yield of crude mitochondria is typically 200–250 μg of mitochondria from 1 g (wet weight) of yeast.

### Mitochondrial ultrastructural characterization using cryo-electron tomography

Freshly prepared crude and magnetic bead-purified mitochondria were diluted to a concentration of ~1 mg/ml in a suspension of BSA-coated 20-nm gold beads (Sigma-Aldrich, St. Louis, MO) in SEH buffer. Samples were then applied to Quantifoil R 2/2 200-mesh copper electron microscopy grids coated with holey carbon (Electron Microscopy Sciences, Hatfield, PA), incubated for 30 sec at RT, and then plunge-frozen in liquid ethane using a Vitrobot Mark IV (FEI, Hillsboro, OR) using a blot force of 2 units and blot time of 8 sec with Whatman # 1 filter paper at 100% humidity at 22°C. Imaging was conducted on a Tecnai F30 Polara (FEI, Hillsboro, OR) microscope operating at 300 kV, with a Gatan K2 Summit electron detection (DED) camera (Gatan, Pleasanton, CA) in super resolution mode. Images were then binned down 2x to achieve a pixel size of 2.635 Å, using SerialEM version 3.6 [23]. Tilt series were collected using a bidirectional tilt scheme from 0°±60° with an increment of 1.5° and a defocus range of -5.0 to -6.0 μm. All identified mitochondria were imaged and included in subsequent analysis, and representative data were collected from both crude and bead-purified mitochondria. Tomograms were aligned using fiducial markers in IMOD version 4.9 [24, 25], followed by three-dimensional volume reconstruction of 3x-binned tilt series via a simultaneous iterative reconstruction technique (SIRT), using Tomo3D [26]. Volumes were subsequently “rotated” in IMOD to retain chirality while re-assigning the z-axis to that of the electron beam. Manual segmentation of mitochondrial membranes was performed on 3x-binned tomograms using the Amira software package, version 5 (Visualization Sciences Group, FEI, Hillsboro, OR).

### Mitochondrial protein import assay

Mitochondrial protein import was assessed as previously described [1, 27, 28]. ^35^S -methionine radiolabeled protein Su9-DHFR was synthesized using TNT Quick Coupled Transcription/Translation System (SP6 promoter, Promega, Madison, WI) with pSu9-DHFR plasmids (a gift from Dr. Carla Koehler, UCLA) and ^35^S-methionine (EasyTag™ L-[35S]-Methionine, PerkinElmer, Waltham, MA). Import reactions were carried out in 200 μl of import buffer containing 50 μg mitochondria, 2 mM ATP, 2 mM NADH and 7.5 μl of ^35^S-Su9-DHFR synthesized as described above for 10 min at 25°C [1]. Valinomycin was added to some import reactions to 5 μg/ml to dissipate mitochondrial Δψ. To remove non-imported precursor, reactions were treated with proteinase K (0.125 mg/ml) for 30 min at 4°C, followed by incubation with PMSF (2.5 mM) for 10 min at 4°C. Mitochondria in all import reactions were concentrated by centrifugation (12,000 x g for 5 min), solubilized in SDS sample buffer, and subjected to SDS-PAGE.

Proteins in polyacrylamide gels were fixed by incubation in a solution consisting of 50% methanol and 5% acetic acid for 30 min. The gel was then washed with water for 30 min to remove acid and then incubated with 1M sodium salicylate for 30 min. The gel was dried using DryEase™ Mini-Gel Drying System (Thermo Scientfic, Grand Island, NY) and was directly exposed to an X-ray film (Carestream® Kodak® BioMax® MR film, Rochester, NY) for 6 hr at -80°C.

### Cytochrome c oxidase activity assay

Cytochrome c Oxidase (COX) activity was assayed as described [29]. 50 μl of crude or bead-purified mitochondria (1–2 mg/ml) were permeabilized with equal volume of 250 mM lauryl maltoside. Cytochrome c (Millipore Sigma, Burlington, MA) was suspended to 1% in 10 mM potassium phosphate buffer pH 7.4 and incubated with the reducing agent sodium dithionite at RT until the color of the solution changed from dark red to orange. The permeabilized mitochondria were added into 1 ml buffer containing 10 mM potassium phosphate and 0.08% cytochrome c freshly reduced by sodium dithionite. COX activity was monitored by following the decrease of absorbance at 550 nm, and expressed in micromoles of cytochrome c oxidized (Cox)/minute/milligram of mitochondrial protein. Cox was calculated from the Beer-Lambert law equation: Cox = ΔA_550_ / (ε_550_ x l), where ΔA_550_ is the change in absorbance, l is the cell path-length, and ε_550_ is the extinction coefficient (ε_550_ = 19.6/mM/cm).

### Western blotting

Western blot analysis was performed as described previously [30]. For analysis of protein fractionation during mitochondrial preparation, 50 μg of protein lysate was loaded onto a 10% SDS-PAGE gel containing 0.5% trichloroethanol (TCE). Prior to transfer, the gel was exposed to UV light (300 nm) for 2.5 min to activate protein-crosslinking activity of TCE [31]. TCE-crosslinked proteins, which were used as load controls, were detected by exposure of gels to 300 nm illumination for 3 sec using a ChemiDoc™ MP Imaging System (Bio-Rad, Hercules, CA). Proteins in blots were detected using Luminata Forte Western HRP substrate (MilliporeSigma, Burlington, MA) and the ChemiDoc™ MP Imaging System (Bio-Rad, Hercules, CA). The primary antibodies used in these studies were rabbit polyclonal antibodies raised against Tom70, cytochrome b2 (gifts from Dr. G. Schatz, University of Basel, Basel, Switzerland), porin (generously provided by Dr. Carla Koehler, UCLA), Sec61 (generously provided by Dr. Randy Schekman, University of California Berkeley), Nyv1 (generously provided by Dr. William Wickner, Dartmouth College, Hanover, NH) and hexokinase (LS-C59302, Lifespan BioSciences, Seattle, WA).

### Statistics

All statistical analysis was performed using GraphPad Prism 7 software. All error bars in graphs indicate mean ± SEM. Statistical analysis of growth rate and COX activity was performed using two-tailed t-tests. Changes in mito-roGFP ratio were analyzed using the Kruskal-Wallis test with Dunn’s multiple comparisons test. The statistical significance of mitochondrial redox asymmetry between buds and mother cells was determined using the Wilcoxon matched-pairs signed rank test. For the comparison of mitochondrial purity and integrity, the significance was determined using one-way ANOVA with Sidak’s multiple comparisons test. In all cases, at least three independent experiments were performed.

## Results and discussion

### Tagging Tom70 with 6xHis has no effect on the fitness of mitochondria or whole cells

**Fig 2:**
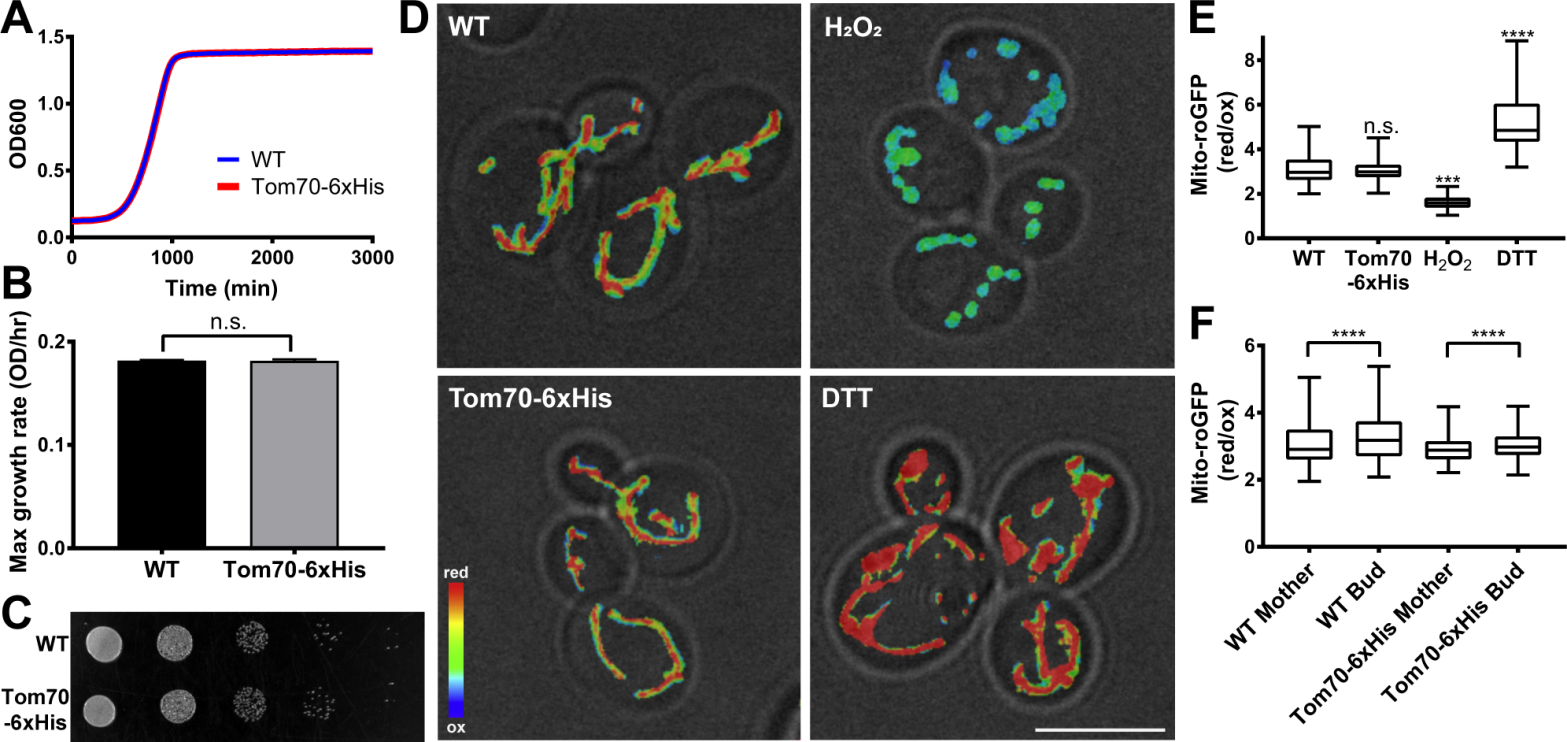
Cellular and mitochondrial fitness are not affected by tagging Tom70 with 6xHis. (A) The growth rate of yeast expressing untagged or 6xHis-tagged Tom70 was monitored by measuring the optical density of the culture in YPD medium at 600 nm (OD_600_) every 20 min for 3 days. (B) Maximum growth rate was calculated from the maximum slope of the growth curve in mid-log phase. There was no significant difference in growth rate of yeast expressing untagged or 6xHis-tagged Tom70 (two-tailed t-test). (C) The respiration-driven growth of yeast expressing untagged or 6xHis-tagged Tom70. Yeast were spotted in 10-fold serial dilutions to YPG plates and incubated at 30°C for 5–7 days. (D) Representative images of mito-roGFP1 in wild-type (WT), Tom70-6xHis cells, and cells treated with 5 mM H_2_O_2_ or DTT. The ratio of the reduced to oxidized roGFP signals is shown in heat maps superimposed on a transmitted-light image. Warmer colors represent more reducing enviroments and cooler colors represent more oxidizing environments. Scale bar = 5 μm. (E) Quantification of mitochondrial redox state using mito-roGFP. H_2_O_2_ and DTT treatments generate highly oxidized and reduced mito-roGFP1, respectively, and illustrate the dynamic range of the sensor. No significant difference was detected between wild-type and Tom70-6xHis cells (Kruskal-Wallis test with Dunn’s multiple comparisons test). Number of cells analyzed: WT = 160; Tom70-6xHis = 139; H_2_O_2_ = 173; DTT = 160. (F) Quantification of mitochondrial redox asymmetry between bud and mother cells of wild type and Tom70-6xHis cells. Buds have a more reduced mitochondrial redox state compared to mother cells in both wild-type and Tom70-6xHis cells (Wilcoxon matched-pairs signed rank test, ****p < 0.0001). Number of cells analyzed: WT = 154; Tom70-6xHis = 130.

Nickel (Ni^2+^) or cobalt (Co^2+^) metal ions have been used to isolate recombinant proteins that are tagged with multiple histidine (His) residues. We applied this concept to isolate mitochondria. First, 6xHis was inserted into the C-terminus of the mitochondrial outer membrane protein Tom70 at its chromosomal locus. The growth rate of yeast expressing Tom70-6xHis is indistinguishable from that of cells with untagged Tom70 in rich, glucose-based media (YPD) (Fig 2A and 2B). Respiration-driven growth of these strains was assessed by growth on solid media containing glycerol (YPG), a non-fermentable carbon source. The respiration-driven growth rates of yeast expressing untagged Tom70 and Tom70-6xHis are also comparable (Fig 2C). Thus, overall cellular fitness is not disturbed by tagging Tom70.

Since Tom70 is a component of the mitochondrial protein import machinery, we also used the redox state of the organelle as a readout for mitochondrial fitness using a mitochondria-targeted ratiometric biosensor, mito-roGFP1 [22, 32, 33]. The dynamic range of the oxidized and reduced states of mito-roGFP1 in cells was demonstrated by treatment with 5 mM hydrogen peroxide (H_2_O_2_) and DTT, respectively (Fig 2D and 2E). We do not detect any significant difference in the overall redox state of mitochondria in yeast expressing tagged and untagged Tom70 (Fig 2D and 2E).

In addition, our previous studies revealed that yeast daughter cells inherit fitter mitochondria that are more reduced compared to mitochondria that are retained in mother cells, and that this asymmetric inheritance of mitochondria affects the fitness and lifespan of both mother and daughter cells [20, 21, 34]. Using mito-roGFP1, we find that tagging of Tom70 with 6xHis has no effect on the asymmetry of mitochondrial redox state (Fig 2D and 2F). Overall, we conclude that tagging Tom70 with 6xHis has no effect on the fitness of mitochondria or the yeast that harbor them.

### Mitochondria isolated using magnetic beads have less contamination

**Fig 3:**
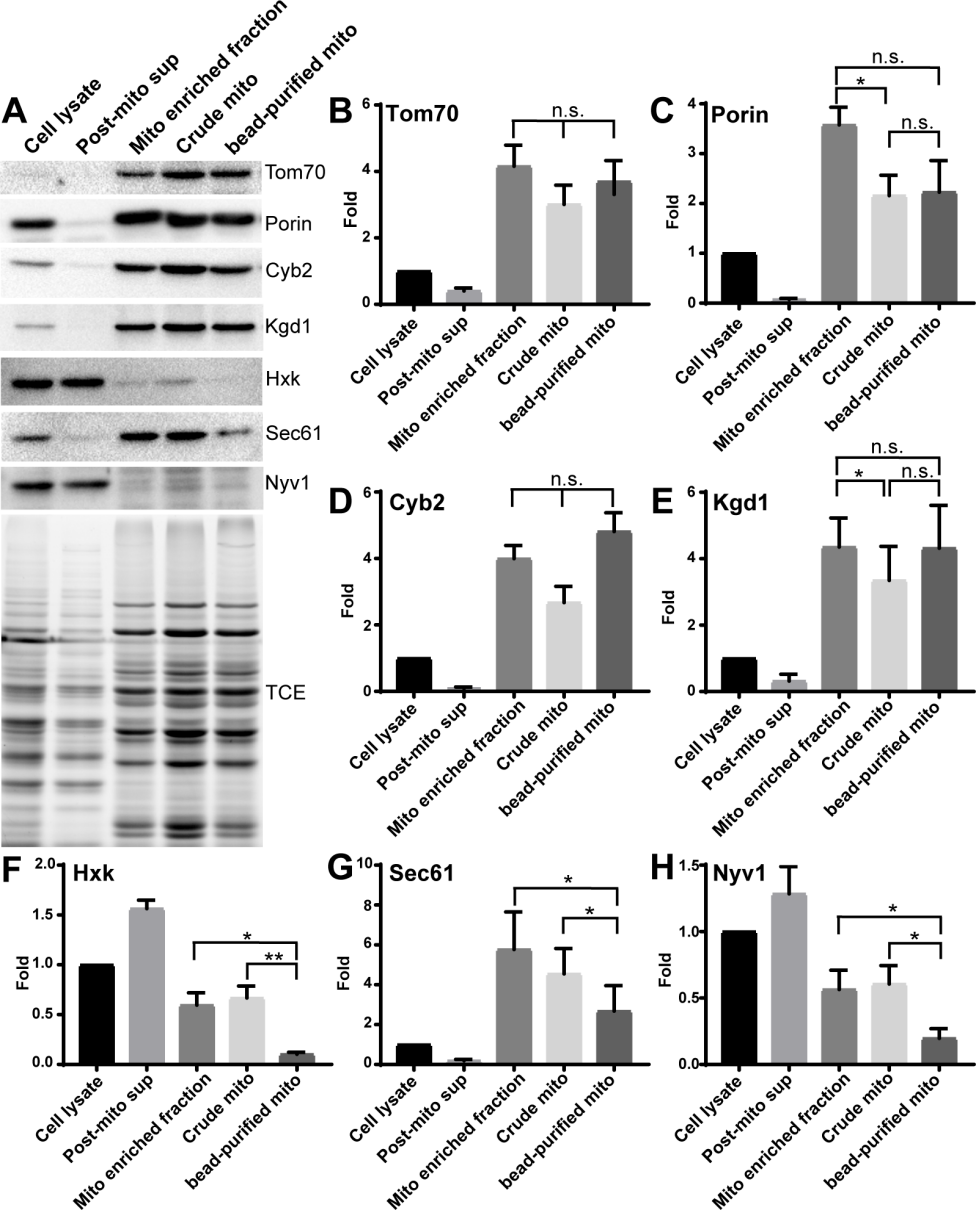
Magnetic bead-purified mitochondria have less contamination compared to crude mitochondria. (A) Tom70, Porin, cytochrome b2 (Cyb2), ketoglutarate dehydrogenase (Kgd1), hexokinase (Hxk), Sec61, and Nyv1 were probed using Western blot analysis of whole-cell lysate, post-mitochondrial supernatant, mitochondria-enriched fraction, crude mitochondria, and magnetic bead-purified mitochondria. Tom70, Porin, Cyb2, and Kgd1 are markers for mitochondrial outer membrane, intermembrane space, and matrix, respectively. Hxk, Sec61 and Nyv1 are markers for cytosol, ER, and vacuole, respectively. Total protein load was assessed using TCE. (B—H) Quantification of fold enrichment of marker proteins shown in (A). Mitochondrial proteins are enriched in bead-purified mitochondria compared to cell lysate, and ER, cytosol, and vacuole contaminants are reduced compared to crude mitochondria (one-way ANOVA with Sidak’s multiple comparisons test). Results shown are representative of 6 trials.

Since crude mitochondria prepared by differential centrifugation are contaminated with proteins and membranes from other organelles, we carried out Western blot analysis using antibodies that recognize marker proteins for mitochondrial outer membrane (Tom70 and porin), intermembrane space (cytochrome b2, Cyb2), matrix (α-ketoglutarate dehydrogenase, Kgd1), ER (Sec61), cytosol (hexokinase), and vacuole (the vSNARE Nyv1) to compare the purity of mitochondrial fractions isolated by differential centrifugation and magnetic beads. As expected, the levels of Tom70, porin, Cyb2, and Kgd1 are enriched in crude or magnetic bead-purified mitochondria compared to whole-cell lysates (Fig 3A–3E) and depleted from the post-mitochondrial fractions. On the other hand, analysis of the fractionation behavior of hexokinase, Sec61, and Nyv1 revealed that mitochondria isolated using magnetic beads have much less cytosolic, ER, and vacuole contamination compared to mitochondria prepared by differential centrifugation (Fig 3A, 3F–3H). Thus, mitochondria isolated using the magnetic bead method are purer compared to crude mitochondria prepared by differential centrifugation.

### Ultrastructure of crude and magnetic bead-purified mitochondria are comparable

**Fig 4:**
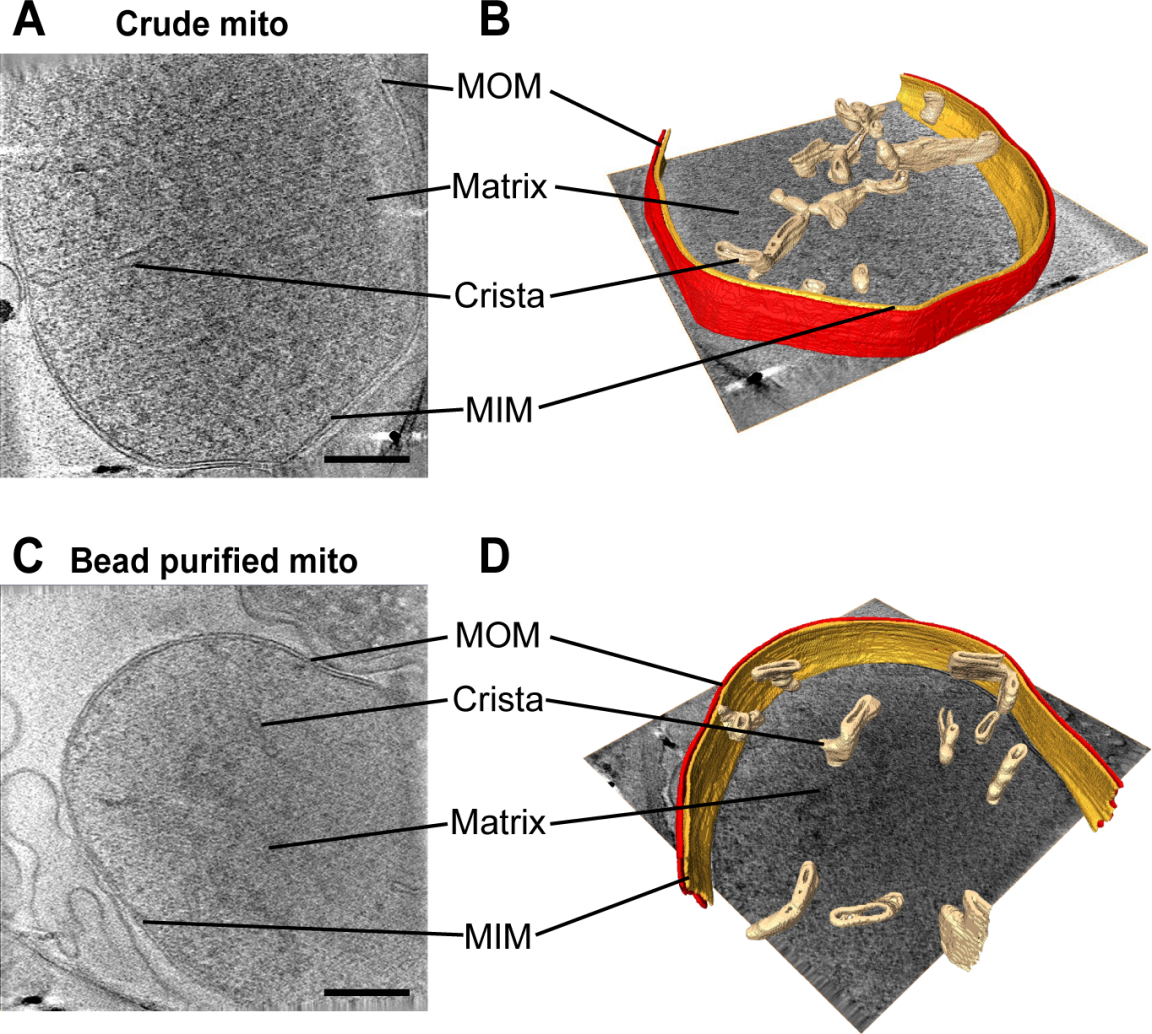
Cryo-electron tomographic analysis of mitochondria reveals preserved ultrastructure. (A and B) Crude and (C and D) magnetic bead-purified mitochondrial preparations were analyzed by cryo-electron tomography. Representative mitochondria are shown. Two-dimensional slices were taken through the middle of the tomographic volumes (left), and three-dimensional models (right) were generated by manual segmentation. Key mitochondrial structural features are indicated, including mitochondrial outer membrane (MOM), crista, matrix, and mitochondrial inner membrane (MIM). Scale bars = 200 nm.

To determine whether mitochondria isolated using magnetic beads preserve their ultrastructure, mitochondria prepared by differential centrifugation or magnetic beads were imaged by cryo-electron tomography, which enables three-dimensional ultrastructural analysis of intact, vitrified organelles without the addition of fixatives or heavy metal contrast agents that can distort and disrupt membranes [35]. In both preparations, mitochondria appeared as circular or oblong double-membrane structures of 500–2,000 nm in apparent diameter (data not shown). Tomograms of representative mitochondria from each preparation revealed intact and closely juxtaposed mitochondrial inner and outer membranes (Fig 4A and 4C). Analysis of crista architecture in 3D models generated from these tomograms revealed predominantly lamellar cristae distributed throughout the matrix in mitochondria from both preparations (Fig 4B and 4D), which is consistent with previous reports [36, 37]. Moreover, these lamellar cristae are indistinguishable between crude and magnetic bead-purified mitochondria. Specifically, the width of cristae of crude and bead-purified mitochondria are 39±3 nm and 40±4 nm, respectively and the length of cristae in crude and bead-purified mitochondria are 175±104 nm and 143±38 nm, respectively. Thus, the magnetic bead method and differential centrifugation both produce mitochondria with similar ultrastructure.

### Mitochondria isolated using magnetic beads are intact

**Fig 5:**
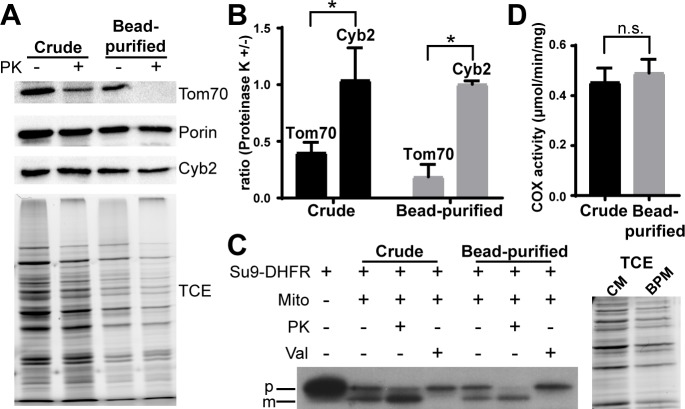
Magnetic bead-purified mitochondria are intact and have robust COX and mitochondrial protein import activity. (A) Crude or bead-purified mitochondria were incubated in the presence or absence of proteinase K (PK, 100 μg/ml) for 30 min at 4°C. Levels of mitochondrial outer membrane proteins (Tom70 and Porin) and an intermembrane space protein (Cyb2) were determined using Western blot analysis. A representative blot is shown. (B) Quantification of the experiment shown in (A). For each purification method, the amount of the indicated protein remaining after proteinase K treatment is shown, normalized to the level in the untreated sample (one-way ANOVA with Sidak’s multiple comparisons test). (C) Import of ^35^S radiolabeled Su9-DHFR into crude and bead-purified mitochondra was carried out as described in Materials and Methods. TCE staining of 10% of the crude and bead-purified mitochondria is shown as a loading control. PK, proteinase K; Val, valinomycin; p, precursor protein; m, mature protein; CM, crude mitochondria; BPM, bead-purified mitochondria. (D) COX activity was measured as the decrease in absorbance at 550 nm resulting from the oxidation of reduced cytochrom c and expressed in micromoles of cytochrome c oxidized/minute/milligram of mitochondrial protein (two-tailed t-test).

To characterize the integrity of mitochondria prepared by the two methods, isolated mitochondria were treated with proteinase K. If isolated organelles are intact, then protease treatment should degrade Tom70, a protease-sensitive outer membrane protein, but should not affect the intermembrane space protein cytochrome b2 (Cyb2). Since the outer membrane protein porin is protease-resistant [38], the level of porin served as a control for protein load in these studies. We find that proteinase K treatment decreased Tom70 levels but not Cyb2 levels in crude and magnetic bead-purified mitochondria (Fig 5A and 5B). Therefore, the integrity of mitochondria prepared by differential centrifugation and magnetic bead affinity purification is similar.

### Mitochondria isolated with magnetic beads have COX and mitochondrial protein import activity

Finally, we characterized whether the isolated mitochondria were functional. We assessed import of Su9-DHFR, a protein consisting of the first 69 residues and the mitochondrial targeting sequence of subunit 9 of the ATPase of *Neurospora crassa* fused to mouse dihydrofolate reductase [39], into mitochondria prepared using both methods. Two forms of Su9-DHFR were recovered with crude and bead-purified mitochondria after import in the cell-free system: one form that is similar in size to full-length Su9-DHFR and another that is smaller than full-length Su9-DHFR (Fig 5C). Treatment with low levels of protease that are sufficient to degrade mitochondrial surface proteins without affecting the integrity of the organelle revealed that full-length Su9-DHFR was degraded and therefore on the surface of the organelle. On the other hand, the small form was protease-protected and therefore within the organelle (Fig 5C, Lane 3 and 6). Finally, in mitochondria treated with valinomycin, which abolishes mitochondrial Δψ, only full-length Su9-DHFR is associated with the organelle (Fig 5C, Lane 4 and 7). These findings indicate that mitochondria prepared using either method have mitochondrial protein import activity: they bind to Su9-DHFR and exhibit Δψ-dependent translocation of the protein across mitochondrial membranes and processing of Su9-DHFR to its mature form [1, 27, 28]. Equally important, the levels of the imported mature form of Su9-DHFR were comparable after normalization to total mitochondrial protein assessed by TCE binding.

In addition, we measured COX activity using a widely used assay. Crude and bead-purified mitochondria have similar COX activity (0.454 ± 0.150 and 0.491 ± 0.145 μmol/min/mg, respectively) (Fig 5D). Thus, our data indicate that mitochondria isolated using magnetic beads are both intact and functional.

## Conclusions

In this study, we developed an easy and quick method to isolate mitochondria from large or small amounts of starting materials. These magnetic bead-purified mitochondria contain less cytosolic, ER, and vacuole contamination but exhibit similar integrity, ultrastructure, mitochondrial protein import and COX activity compared to crude mitochondria prepared by differential centrifugation. Since the mitochondria are tagged with 6xHis in an endogenous mitochondrial outer membrane protein and purified using commercially available Ni-NTA magnetic beads, this approach is more cost-effective compared to antibody-based magnetic bead methods and enables release of mitochondria from the magnetic beads, as needed. In addition, because endogenous tagging in mammalian systems is now more tractable using CRISPR [40–42], our method is not only limited to the yeast system, but is also suited for use in mammalian cells or any other model system.
